# Low Starch/Low Dairy Diet Results in Successful Treatment of Obesity and Co-Morbidities Linked to Polycystic Ovary Syndrome (PCOS)

**DOI:** 10.4172/2165-7904.1000259

**Published:** 2015-04-30

**Authors:** Jennifer L. Phy, Ali M. Pohlmeier, Jamie A. Cooper, Phillip Watkins, Julian Spallholz, Kitty S. Harris, Abbey B. Berenson, Mallory Boylan

**Affiliations:** 1 Department of OB-GYN, Center for Fertility & Reproductive Surgery, Texas Tech University Health Sciences Center, Lubbock, TX, USA; 2 Department of Nutritional Sciences, Texas Tech University, Lubbock, TX, USA; 3 Department of OB-GYN, Center for Interdisciplinary Research in Women’s Health, University of Texas Medical Branch, Galveston, TX, USA; 4 Clinical Research Institute, Department of Statistics, Texas Tech University Health Sciences Center, Lubbock, TX, USA; 5 Center for the Study of Addiction and Recovery, Texas Tech University, Lubbock, TX, USA

**Keywords:** PCOS, Diet, Hyperinsulinemia, Insulinemic, Insulinogenic, Weight Loss

## Abstract

**Background:**

Polycystic Ovary Syndrome (PCOS) affects approximately 15% of reproductive-age women and increases risk of insulin resistance, type 2 diabetes mellitus, cardiovascular disease, cancer and infertility. Hyperinsulinemia is believed to contribute to or worsen all of these conditions, and increases androgens in women with PCOS. Carbohydrates are the main stimulators of insulin release, but research shows that dairy products and starches elicit greater postprandial insulin secretion than non-starchy vegetables and fruits. The purpose of this study was to determine whether an 8-week low-starch/low-dairy diet results in weight loss, increased insulin sensitivity, and reduced testosterone in women with PCOS.

**Methods:**

Prospective 8-week dietary intervention using an ad libitum low starch/low dairy diet in 24 overweight and obese women (BMI ≥ 25 kg/m^2^ and ≤ 45 kg/m^2^) with PCOS. Diagnosis of PCOS was based on the Rotterdam criteria. Weight, BMI, Waist Circumference (WC), Waist-to-Height Ratio (WHtR), fasting and 2-hour glucose and insulin, homeostasis model assessment of Insulin Resistance (HOMA-IR), HbA1c, total and free testosterone, and Ferriman-Gallwey scores were measured before and after the 8-week intervention.

**Results:**

There was a reduction in weight (−8.61 ± 2.34 kg, p<0.001), BMI (−3.25 ± 0.88 kg/m^2^, p<0.001), WC (−8.4 ± 3.1 cm, p<0.001), WHtR (−0.05 ± 0.02 inches, p<0.001), fasting insulin (−17.0 ± 13.6 μg/mL, p<0.001) and 2-hour insulin (−82.8 ± 177.7 μg/mL, p=0.03), and HOMA-IR (−1.9 ± 1.2, p<0.001) after diet intervention. Total testosterone (−10.0 ± 17.0 ng/dL, p=0.008), free testosterone (−1.8 pg/dL, p=0.043) and Ferriman-Gallwey scores (−2.1 ± 2.7 points (p=0.001) were also reduced from pre- to post-intervention.

**Conclusion:**

An 8-week low-starch/low-dairy diet resulted in weight loss, improved insulin sensitivity and reduced testosterone in women with PCOS.

## Introduction

Polycystic Ovary Syndrome (PCOS) is a condition of hyperandrogenism and affects approximately 1 in 8 women of reproductive-aged worldwide [1]. Women with PCOS often suffer from obesity, insulin resistance, and clinical symptoms of elevated androgens, such as hirsutism, acne, irregular menses, and abdominal obesity. Prolonged or high degrees of postprandial hyperinsulinemia are believed to contribute to or worsen all of these conditions [2–5]. Further, insulin resistance and hyperinsulinemia increase androgen levels in women with PCOS and may make weight loss difficult [2,3,6].

Insulin is the primary hormonal mediator of energy storage [7], and while hyperinsulinemia is common in PCOS, it is unclear whether it is due to excessive insulin secretion, decreased insulin clearance, or both [8]. Persistently elevated insulin levels, immaterial of its origin, can result in insensitivity of its target cells over time, which further exacerbates hyperinsulinemia and increases the potential for beta cell dysfunction [9]. Research has shown that methods which limit or reduce peripheral hyperinsulinemia in insulin resistant individuals have the potential to treat or prevent obesity and related disease [3,10]. This may be due to the reduced ability of insulin-resistant individuals to oxidize fatty acids because of elevated insulin levels, leading to tissue accumulation of triglycerides in skeletal muscle and further impaired insulin signaling [11,12]. Hyperinsulinemia decreases expression of Lipoprotein Lipase (LPL), an enzyme responsible for lipolysis, in skeletal muscle, but increases LPL expression in adipose tissue. High LPL activity in the adipose tissue coupled with low LPL activity in skeletal muscle drives lipolysis in the adipocyte, resulting in high levels of circulating fatty acids that further impair insulin signaling and lipid oxidation in skeletal muscle [13]. This reduced activity of LPL in skeletal muscle, and subsequently reduced lipid oxidation, has been linked with weight gain in prospective studies [11,14,15]. Therefore, reduced utilization of lipid as an energy substrate has the potential to further enhance metabolic dysfunction [12].

A recent study conducted by Mehran et al. [3] used mice lacking the Ins1 gene, which contributes to approximately 30% of secreted insulin, to determine whether these mice would be incapable of high-fat diet induced obesity. The Ins1 deficient mice were protected from diet induced weight gain, suggesting that pancreatic hyper-secretion of insulin is required for diet-induced obesity. Another study found that pharmacological suppression of insulin secretion in obese adults consuming an ad libitum diet resulted in weight loss and reduced intake of, and preference for, carbohydrate-rich foods [16]. Therefore, novel methods that lead to a reduction in hyperinsulinemia may have important implications for facilitating weight loss, improving insulin resistance and lowering androgens in women with PCOS [17].

Studies indicate that there is dissociation between the glycemic response and insulinemic response in some carbohydrate foods [4,18,19]; this dissociation may be even greater in insulin resistant populations [20]. Starches, dairy foods and those with added sugars have shown to have an increased insulin response compared to non-starchy vegetables and fruit [21–27]. Therefore, the purpose of this study was to determine whether removing starches and dairy for 8 weeks because of their insulinemic properties would result in weight loss, increased insulin sensitivity, and reduced testosterone in adult women with PCOS. We hypothesized that an 8-week low starch/low dairy diet would lead to weight loss and improved body composition, increased insulin sensitivity, and reduced testosterone in women with PCOS.

## Methods

### Study participants

Twenty-eight women (BMI ≥ 25 kg/m^2^ and ≤ 45 kg/m^2^) with a confirmed diagnosis of PCOS were recruited from a gynecological/obstetrical and fertility clinic under the supervision of a Reproductive Endocrinologist (REI). Eligible women were between 18–45 years of age with a BMI ≥ 25 kg/m^2^ and ≤ 45 kg/m^2^. Diagnosis of PCOS was based on oligo- and/or amenorrhea and the presence of hyperandrogenism (clinical and/or biochemical), consistent with the Rotterdam criteria (2004). All participants had at least one polycystic ovary by ultrasound. Oligomenorrhea was determined by cycle length (>35 days), and amenorrhea was determined as lack of a menstrual period ≥ 12 months. Clinical hyperandrogenism (hirsutism, severe acne, or androgenic alopecia) and/or biochemical hyperandrogenism (testosterone >55 ng/dl) was assessed by the REI. Women with adrenal enzyme defects such as Cushing’s Syndrome or adrenal virilizing tumors, participants with Type 2 Diabetes (T2D), evidence of late onset 21-hydroxylase deficiency, or any other medical condition requiring supervision were excluded from the study. Women who were nursing during the length of the study, women with a confirmed eating disorder, and women with gastrointestinal absorption issues were excluded as well. Subjects discontinued insulin sensitizers, oral contraceptives, and cyclic progesterone for one month prior to the study. This study was approved by the Institutional Review Board of Texas Tech University Health Sciences Center and written informed consent was obtained from all participants prior to beginning the study.

### Protocol

Weight, height, Waist Circumference (WC), Waist-to-Height Ratio (WHtR), and body fat percentage were measured on week 0 and week 8. BMI was calculated using weight and height. The BODPOD (Cosmed Chicago, IL) was used to measure body weight and body composition. Fasting and 2-hour serum glucose and insulin levels were measured via a 75 g 2-hr Oral Glucose Tolerance Test (OGTT) with blood samples taken at 0 and 120 minutes at week 0 and week 8. HemoglobinA1c (HbA1c), blood lipids, total and free testosterone, and 25-OH vitamin D were measured via a fasting blood sample at week 0 and week 8. Modified Ferriman-Gallwey scores, a method of evaluating and quantifying hirsutism, were calculated by the REI [28]. To excludes the possibility of seasonal variations of sun exposure and natural fluctuations of 25-OH vitamin D, 50% of the participants began the study in the summer and early fall months and concluded in the late fall and winter months.

### Intervention

Subjects were instructed to follow a low starch/low dairy diet throughout the 8-week study. Each participant spent 2 hours with a Registered Dietitian (AMP) for intensive diet education. Each participant was provided with written materials that included appropriate foods and products, example meal plans, a guide to eating out, and recipes. After initial instruction, subjects did not have face-to-face contact with the Registered Dietitian until they returned to the clinic for measurements on week 8. Participants were instructed to eat lean animal protein (meat and poultry), fish and shellfish, eggs, non-starchy vegetables, low-sugar fruits (berries, apples, oranges, plums, etc.), avocado, olives, nuts and seeds, and oils (olive and coconut). Subjects older than 21 years were allowed 6 oz. of red wine per day, and all subjects were allowed up to 1oz of prepared or fresh, full-fat cheese per day. Previous studies have shown cheese to be less insulinemic than other dairy products, thus, cheese was allowed in restricted amounts to aid in dietary compliance. The diet excluded all grains, beans, other dairy products, and sugar (including fruit juice from concentrate, raw turbinado sugar, evaporated cane juice, high-fructose corn syrup, honey, or agave nectar) because of their insulinemic properties. Non-nutritive sugar substitutes were allowed for participants that wished to use them. Green vegetables, nuts, and seeds are good sources of calcium, but calcium-fortified non-dairy alternatives (unsweetened almond milk and unsweetened coconut milk) were allowed for participants that wished to supplement their calcium intake. Participants were not advised to count calories or carbohydrates and were encouraged to eat until they were satisfied, but not to overeat. Participants were instructed not to change their level of physical activity throughout the intervention. Three day food records (Thursday, Friday and Saturday) were collected at weeks 1, 4, and 7 to determine dietary compliance.

### Assays

Glucose, insulin, and HbA1c were run by one laboratory (University Medical Center, Lubbock, TX), and testosterone and free testosterone assays were run by another laboratory (Quest Diagnostics Nichols Institute, San Juan Capistrano, CA). All assays were performed on the Roche Diagnostics Cobas 6000. Glucose was performed by enzymatic reference method with hexokinase, HbA1c by turbidimetric inhibition immunoassay, insulin by electrochemiluminescence immunoassay, and testosterone by electrochemiluminescence immunoassay. The Homeostasis Model Assessment (HOMA) was used as a surrogate measure of insulin resistance and was calculated using the standard equation [29]. Cholesterol was assayed by enzymatic colorimetric test and 25-OH vitamin D by electrochemiluminescence binding assay. Free testosterone was calculated taking the concentrations of total testosterone and Sex Hormone Binding Globulin (SHBG) into account and assuming a fixed albumin concentration of 43 g/L, as described elsewhere (Vermeulen et al. 1999).

### Statistical analysis

As pre- and post-diet measurements were taken for all outcome variables, paired t-tests were used to determine statistically significant changes. Some of the outcomes of interest were significantly non-normally distributed. However, as the paired t-test is robust against the violation of the assumption of normality and N=24 is a relatively large sample size for paired testing procedures, we only departed from the parametric t-test in cases of extreme outlying observations (such as 2 hr insulin>1000 μg/mL). Statistical analysis was performed using SAS 9.3 (SAS Institute, Cary NC).

## Results

Of the 28 women recruited, 24 completed the study (3 dropped out due to non-compliance and 1 spontaneously conceived during the study). The average age of participants was 29.8 ± 4.0 years. Thirteen of the participants were Caucasian, 9 were Hispanic, 1 was Pacific Islander and 1 was Native American. Dietary intake data can be found in Table 1.

There was a significant reduction in all anthropometric measurements after diet intervention (Table 2). While fasting glucose was significantly reduced (p=.002), 2-hour glucose did not appear to change significantly (p=0.657) between the pre and post diet states. There was a small but significant reduction in HbA1c (p=0.001), though the clinical significance of a decrease of −0.25 ± 0.32% may be debatable. Fasting insulin, 2-hour insulin and HOMA-IR were reduced by 52%, 37% and 51%, respectively. In addition, free and total testosterone was reduced by 23% and 19%, respectively. There was a slight reduction in Ferriman-Gallwey scores (−2.1 ± 2.7, p=0.001) after the 8-week intervention.

Several blood lipids were significantly reduced. Triglycerides and VLDL cholesterol were reduced by 35% and 29%, respectively. HDL cholesterol (−5.7 ± 9.1 mg/dl, p=0.006) was also significantly reduced, but changes in total cholesterol and LDL cholesterol were not significant.

While there was a reduction in HDL, change in the ratio of total cholesterol/HDL was not significant after the 8-week diet. Mean 25-OH vitamin D levels increased 22% from pre- to post-study without supplementation or increased sun exposure.

## Discussion

These findings show that an 8-week low starch/low dairy diet resulted in weight loss, increased insulin sensitivity, and reduced free and total testosterone in women with PCOS. Several additional outcome measures were unexpectedly improved, including a reduction in VLDL and triglycerides, and an increase in vitamin D levels. Considering the dietary intervention spanned only 8 weeks with no additional exercise recommendations, medications, or supplements, these improvements are both highly clinically and statistically significant. In addition, this dietary approached provides a possible alternative to metformin for patients with impaired glucose tolerance who struggle with gastrointestinal side effects or who are noncompliant.

The women in the current study showed a significant reduction in body weight, body fat percentage and waist circumference, which could be attributed to the relatively low energy and/or carbohydrate intake. The anthropometric and biochemical improvements shown here are consistent with other studies utilizing a reduced carbohydrate diet for the treatment of PCOS [30,31]; however, these studies required participants to keep carbohydrates to <20 g per day, which may be unsustainable for many individuals. Studies have also utilized a low glycemic index (GI) diet in the treatment of PCOS [31–34]. A low GI diet focuses on foods that do not cause a spike in blood sugar to prevent a subsequent spike in insulin. However, neither postprandial insulin secretion nor insulin resistance was taken into consideration when developing the glycemic index. Therefore, a low GI diet may not be ideal for a PCOS population because of the insulinemic effects of some low glycemic foods [4,17,20,35]. One study using an ad libitum low GI diet for the treatment of PCOS followed patients until they lost 7% of their baseline body weight, or approximately one year [33]. The participants in the current study lost over 8% of their baseline bodyweight in only 8 weeks and achieved greater improvements in biochemical outcomes. We did not, however, measure long-term sustainability, so it remains unknown whether this diet is sustainable over time. Future studies are needed to determine the long-term sustainability of this diet and to compare anthropometric and biochemical outcomes after a low insulinemic diet compared to an isocaloric low GI diet in women with PCOS.

While the improvements in markers of insulin resistance and hyperinsulinemia may be related to weight loss in our study participants, some studies [36–38], but not all [39], have found that weight loss may be more difficult in women with PCOS, which may be related insulin resistance. Hyperinsulinemia is strongly associated with obesity, but whether insulin drives obesity or is simply a compensatory response to obesity-driven insulin resistance remains unknown [3,40]. Studies have shown that diet-induced hyperinsulinemia via the consumption of a high glycemic/high insulinemic diet promotes obesity, insulin resistance, and related disease [3,5,10,40,41], as well as carbohydrate craving [42] and decreased fat oxidation [12]. Further, one study found that pharmacological suppression of hyperinsulinemia in obese adults consuming and ad libitum diet resulted in weight loss and reduced carbohydrate intake [16]. The results of the current study demonstrate that reduction in insulinemic foods (starches, dairy products, and added sugars) leads to weight loss and increased insulin sensitivity, as well as reduced testosterone in women with PCOS. The improvements in insulin sensitivity and testosterone could be due to the weight loss, the diet, a reduction in overall energy and/or carbohydrate consumption, or a combination of the above. Considering all subjects had a reduction in weight, and weight and diet are associated, it is difficult to separate the effects of diet and weight loss on insulin sensitivity and testosterone levels.

Considering the distressing clinical symptoms associated with elevated androgens in women with PCOS, the significant decrease in free and total testosterone (Table 2), as well as modified Ferriman-Gallwey scores after the diet intervention and without insulin sensitizing or anti-androgenic medication is very clinically relevant. Lifestyle interventions that promote weight loss have previously been shown to improve insulin and androgen levels in this population [31,33]. The intervention used in this study achieved similar if not further reductions in weight loss and insulin and androgen levels as other lifestyle intervention studies, however, many of those studies were either longer in duration [33,43] or utilized pharmacotherapy as an addition to the lifestyle intervention [44,45]. Further studies are needed to compare a low starch/low dairy diet to other lifestyle and pharmacologic interventions of equal duration in women with PCOS.

The majority of the participants were hoping to conceive following the intervention. Research has shown that maternal pre-pregnancy obesity and insulin resistance increases the risk of adverse pregnancy outcomes, including pre- and post-natal weight gain, preeclampsia, cesarean delivery, congenital abnormalities and large for gestational age infant [46–48], as well as development of obesity and early-onset T2D in the child [49,50]. Therefore, there is a need for evidence-based preconception diet and lifestyle interventions to help women achieve a healthy weight before pregnancy. The women in the current study were encouraged to consume nutrient dense food, such as lean protein, vegetables and fruits, and nuts and seeds, which limits the possibility of nutrient deficiencies; thus, this dietary approach is appropriate for women trying to conceive. Considering the improvements in anthropometric measures and insulin sensitivity in the women in the current study, this dietary approach may be an effective preconception strategy to help overweight and obese women with PCOS reach a healthy weight before pregnancy. Unfortunately, it is out of the scope of this study to make any definitive statements about the ability of this type of diet to improve pregnancy outcomes in these adult women.

The major weakness of this study design was lack of a control group. In this study, the subjects served as their own controls through the utilization of the pre/post testing study design. Future studies are needed to determine if similar results are reproducible in a larger study group in comparison to age-matched, BMI-matched controls ingesting a comparable caloric standard diet or low glycemic index diet. Additionally, we did not measure any satiety hormones or adipokines in this study. Future studies should include analysis of changes in C-peptide, calcium, ghrelin, glucagon like peptide-1, peptide YY, leptin, adiponectin and kisspeptin after the dietary intervention. Additionally, future studies could assess hunger and fullness through a validated Visual Analog Scale (VAS) to determine whether this diet increases subjective satiety.

## Conclusion

In conclusion, an 8-week diet eliminating insulinemic foods resulted in anthropometric improvements including weight loss, a reduction in waist circumference, and body fat loss in an overweight and obese PCOS population. This diet also led to improvements in insulin sensitivity as determined by HOMA-IR and a reduction in total and free testosterone in women with PCOS. Considering this dietary intervention allowed for ad libitum intake without calorie or carbohydrate counting or medications, these improvements are promising, especially since weight loss is difficult in a PCOS population. Further research is needed regarding the reduction or elimination of insulinemic foods as a treatment for hyperinsulinemia and insulin resistance in overweight and obese women with PCOS.

## Figures and Tables

**Table 1:** Nutritional analysis of food logs (n=24).

	Mean ± SD
Energy (kcal)	1422 ± 199
Total Fat (g)	72.1 ± 16.5
MUFA (g)	31.9 ± 9.1
PUFA (g)	15.8 ± 6.8
SFA (g)	19.5 ± 7.5
Carbohydrate (g)	94.3 ± 22.8
Fiber (g)	24.4 ± 6.6
Protein (g)	98.0 ± 25.1

MUFA: Monounsaturated Fat; PUFA: Polyunsaturated Fat; SFA: Saturated Fat

**Table 2:** Change in anthropometric and biochemical outcomes after diet intervention (n=24).

	Baseline	Week 8	Change	
	Mean(±SD)	Mean(±SD)	Mean(±SD)	P value^a^
Weight (kg)	102.1 ± 17.4	93.5 ± 16.9	−8.6 ± 2.3	<0.0001
BMI (kg/m^2^)	38.3 ± 5.5	35.1 ± 5.5	−3.2 ± 0.9	<0.0001
Waist circumference (cm)	109.8 ± 13.1	101.4 ± 12.3	−8.4 ± 3.1	<0.0001
Waist-to-height ratio	0.67 ± 0.08	0.62 ± 0.07	−0.05 ± 0.02	<0.0001
% Fat Mass	46.3 ± 6.8	44.6 ± 4.7	−1.5 ± 2.8	0.02
Glucose fasting (mg/dl)	95.0 ± 19.6	86.0 ± 8.4	−8.9 ± 17.1	0.01
Glucose 120min (mg/dl)	128.0 ± 67.7	114.9 ± 33.7	−13.1 ± 54.3	NS
Insulin fasting (μg/mL)	32.7 ± 17.7	15.7 ± 6.8	−17.0 ± 13.6	<0.0001
Insulin 120min (μg/mL)	225.8 ± 229.4	142.9 ± 93.4	−82.8 ± 177.7	0.03
HOMA-IR	3.9 ± 1.5	1.9 ± 0.9	−1.9 ± 1.2	<0.0001
HgbA1c (%)	5.5 ± 0.4	5.2 ± 0.4	−0.3 ± 0.3	0.001
Total cholesterol (mg/dl)	195.9 ± 27.6	186.7 ± 27.2	−9.3 ± 25.5	0.09
VLDL cholesterol (mg/dl)	32.4 ± 15.8	21.7 ± 6.7	−9.3 ± 13.4	<0.0001
LDL cholesterol (mg/dl)	127.7 ± 25.8	124.7 ± 21.6	−2.3 ± 20.5	NS
HDL cholesterol (mg/dl)	47.6 ± 13.0	41.9 ± 10.1	−5.7 ± 9.1	0.006
Triglycerides (mg/dl)	162.8 ± 79.1	108.2 ± 34.0	−57.0 ± 9.1	<0.0001
Total testosterone (ng/dl)	53.3 ± 24.5	43.3 ± 17.6	−10.0 ± 17.0	0.008
Free testosterone (pg/dl)	7.8 ± 4.8	6.0 ± 2.1	−1.8 ± 3.9	0.04
25-OH Vitamin D (ngl/mL)	20.3 ± 10.9	24.7 ± 12.2	+4.4 ± 6.4	0.003
Ferriman Gallwey Score	11.9 ± 6.8	9.8 ± 6.5	−2.1 ± 2.7	0.007
